# South Asian youth mental health in Peel Region, Canada: Service provider perspectives

**DOI:** 10.1177/13634615221119384

**Published:** 2022-09-13

**Authors:** Farah Islam, Syeda Qasim, Muhanad Ali, Michaela Hynie, Yogendra Shakya, Kwame McKenzie

**Affiliations:** 1 Centre for Addiction and Mental Health, 7938 University of Toronto, Canada; 2 Data and Psychospiritual Department, Yaqeen Institute for Islamic Research Canada, Canada; 3 Department of Education, Ontario International Medical Graduate School, Canada; 4 Department of Health Studies, School of Health Policy and Management, York University, Canada; 5 Department of Public Health, Centre for Refugee Studies, York University, Toronto, Canada; 6 Dalla Lana School of Public Health, 7938 University of Toronto, Canada; 7 Wellesley Institute, Toronto, Canada

**Keywords:** acculturation, Canada, immigrant, mental health, mental health service, newcomer, stress, service provider, South Asian, youth

## Abstract

The Peel Region of Toronto, Canada is home to over a third of the province's South Asian population. Youth are at a vulnerable time period in terms of their mental health. South Asian youth populations may face additional challenges to their mental health such as acculturative stress, intergenerational conflict, and racism and discrimination. This qualitative study set out to understand the mental health concerns and service access barriers experienced by South Asian youth populations in the Peel Region of Toronto, Canada from the perspective of mental health service providers. In-depth semi-structured interviews were carried out with mental health service providers (n  =  22) who work with South Asian youth living in Peel Region. Thematic analysis was used to elucidate themes related to mental health stressors and service access barriers experienced by youth. According to mental health service providers, South Asian youth navigate a number of unique stressors related to the domains of culture, religion, and family dynamics, experiences of discrimination, the impact of migration, beliefs around mental illness and help-seeking, help-seeking trajectories and therapy recommendations, and lastly, sex differences. Mental health service providers outlined steps needed to effectively address the unique mental health challenges, best practice guidelines, and recommendations for working with South Asian youth, families, and communities to provide a practical and nuanced overview on how a multi-level strategy for mental health care can effectively meet the needs of South Asian youth populations.

## Introduction

The South Asian diaspora (e.g., from South Asian countries such as India, Pakistan, Bangladesh, Sri Lanka, or Nepal) numbers at over 30 million people globally (Rai & Reeves, 2008). Major South Asian diaspora communities have settled in regions such as the Arabian Gulf, Uganda, South Africa, the Caribbean, Australia, the UK, the US, and Canada (Rai & Reeves, 2008). Canada experienced a major influx of migration of South Asian populations in the 1960s and 1970s (Statistics Canada, 2005). Currently, close to 2 million people in Canada are of South Asian origin (i.e., with origins in South Asian countries or Indo-Caribbean roots), making up the largest segment of Canada's racialized populations. In Canada, racialized populations refer to “visible minority” or non-white populations (Li, 2003). Close to 60% of the nation's South Asian populations reside in Ontario and over a third (1,150,415) of the province's South Asian populations live in Peel Region, one of five regions making up the larger metropolitan area of the city of Toronto (Region of Peel, 2017; Statistics Canada, 2016, 2017a, 2017b). The Peel Region of the Greater Toronto Area includes the cities of Brampton and Mississauga and the town of Caledon, and has some of the highest percentages of South Asian populations in the Greater Toronto Area (e.g., 44% of the Brampton's population and 23% of Mississauga's population is South Asian) (Statistics Canada, 2019). Close to 15% of people in Brampton speak only Punjabi at home, while Urdu is the top non-official language (not English or French) spoken in Mississauga (3%) (Peel Region, 2012). The majority of South Asian immigrant populations arrived in Peel Region after 1980 and their numbers have been steadily growing (Region of Peel, 2012). Peel has the highest percentage of youth aged 15–24 (14%) in the larger metropolitan area. The region is also growing quickly, with 20,000 new people settling in Peel each year (Region of Peel, 2019, 2017).

As South Asian communities in Peel Region continue to grow, there is a need to effectively address the mental health needs and well-being of these communities, particularly for youth (Mental Health Commission of Canada, 2016a; Region of Peel, 2017). Exposure to a confluence of physical, emotional, and social changes (e.g., violence, trauma, abuse, poverty, etc.) can make youth and young adults vulnerable to mental health issues (World Health Organization, 2020). By the age of 25, one in five Canadians report a mental illness, and 70% report that symptoms first began in childhood (Mental Health Commission of Canada, 2016b). Immigrant youth in particular are exposed to multiple risk factors for mental health problems (Shakya et al., 2010). Linguistic barriers, especially within the education system and labor market, have been linked to mental health challenges facing newcomer youth. Challenges such as income insecurity, unemployment, underemployment, and precarious employment have significant mental health implications for both youth and their families (e.g., depression, family tensions, and other mental health stresses) (Shakya et al., 2010). Settlement stressors related to linguistic barriers, particularly around navigating the Canadian education system (the inability to establish friendships, difficulty following and understanding the educator and curriculum, and bullying), also position adolescents to be more susceptible to experience stress, anxiety, and low self-esteem (Shakya et al., 2010). However, immigrant and refugee youth have some of the lowest rates of mental health service utilization (Saunders et al., 2018), and are more likely to present with their first mental health crisis in the emergency department compared to non-immigrants, which is associated with poorer outcomes and can lead to gaps in continuity of mental health care (Saunders et al., 2018).

Research focusing specifically on South Asian populations in Canada finds that they have lower prevalence rates of mental health service use (Grace et al., 2016; Mental Health Commission of Canada, 2016a; Tiwari & Wang, 2008). Gadalla (2010) found low utilization of mental health services amongst South Asian populations, where almost one-third of South Asians diagnosed with a major depressive episode cited no access to available care and had the lowest possible odds of seeking mental health care compared to other ethnic groups (Gadalla, 2010). This may be the result of a number of barriers that South Asian populations face in accessing mental health services, including inadequate awareness of services, settlement barriers, lack of culturally sensitive approaches, stigma, language barriers, unusually long wait times, and lack of trust in the mental health system (Reitmanova & Gustafson, 2009; Saunders et al., 2018; Thomson et al., 2015).

The Mental Health Commission of Canada (2015) has suggested that improved services for immigrant, refugee, ethnocultural, and racialized communities need to be built across Canada using local data. Yet despite the growing South Asian youth population in Peel Region, the vulnerability of youth in terms of mental health, and the considerable mental health service access barriers experienced by South Asian populations, there is very little research exploring the mental health concerns and service access barriers of South Asian youth populations. Thus, this study sought to carry out a mental health needs assessment of South Asian youth in Peel at a systems level from the perspective of mental health service providers. This study is part of a larger multiphase study to understand the mental health needs of South Asian youth. An already published leg of the study examined interviews with South Asian youth (Islam et al., 2017). Interviews with key informants such as mental health service providers can offer added insight. While youth interviews revealed many gaps within South Asian families and communities, interviews with mental health service providers can offer in-depth perspective gaps within the mental health system. Considering the diverse and intersecting social locations of the South Asian diaspora, coupled with multiple experiences of inequities navigating both the Canadian health care system and society, an intersectional and social determinants of health framework was employed to expand our understanding of mental health inequities and highlight the limitations of the mental health system in order to improve access to mental health services for South Asian youth.

## Methods

### Theoretical frameworks

A social determinants of health approach emphasizes that poorer health and mental health are the result of inequality, rather than poverty. While poor social, material, and physical conditions affect the poorest the most, inequalities in access to power and material resources affect health and well-being such that the risks of poor mental health and mental illness increase as groups experience greater inequalities along the entire social, economic, and political gradient (Allen et al., 2014; Mikkonen & Raphael, 2010).

An intersectional lens (Crenshaw, 1989; Smith, 2011) was applied to examine the ways in which the interconnected and overlapping social locations (ethnicity, sex, class, religion, to name a few) of South Asian youth populations simultaneously intersect and compound within systems, institutions, and processes to produce inequities at both the micro (i.e., interpersonal/intrapersonal) and macro level (structural barriers). These multiple structures of inequalities intersect to produce hierarchical matrices of domination (Hill Collins, 1990). We acknowledge that these identities are both socially constructed and mutually constitutive, shifting our focus towards issues of power, privilege, and discrimination.

### Research design and research questions

This exploratory qualitative study was carried out as part of a larger multiphase study to understand the mental health needs of South Asian youth. This study was designed to answer the following research questions from the perspective of mental health service providers working with South Asian youth populations:

1. What are the unique mental health concerns and mental health service access barriers experienced by South Asian youth?
2. Are there sex differences in mental health stressors and help-seeking patterns?
3. What help-seeking trajectories do South Asian youth take when seeking mental health care?
4. What best practice guidelines and recommendations do mental health service providers offer for working with South Asian youth populations?
5. What recommendations for mental health system-level change do mental health service providers offer?

### Sampling

As very specific criteria (having experience of working in mental health care provision for South Asian youth in Peel Region) was required for this study, purposive snowball sampling was used to make data collection feasible, by first beginning with a small set of individuals selected for their prominence and/or connectedness in the community of providers serving this population and then expanding further through their referrals and recommendations. All participants were assured that participation was voluntary. Care was taken to accommodate their schedule and select convenient meeting locations. Random sampling was also employed through the use of recruitment flyers posted in public areas and sent by email to mainstream (e.g., the Canadian Mental Health Association of Peel and YMCA) and culturally specific mental health organizations (e.g., Punjabi Community Health Services and Sayeda Khadijah Centre, one of the largest mosques in Peel). The recruitment flyer was also posted on social media via Twitter and on the Centre for Addiction and Mental Health's Evidence Exchange Network. Participant interviews took place from March to June 2015 and were conducted at locations convenient for participants.

### Rigor

In order to ensure rigor (Guba & Lincoln, 1981, 1982; Lincoln & Guba, 1985), the recruitment process and interviews were offered in a variety of South Asian languages (Punjabi, Urdu, Hindi, and Bengali) as well as English. The positionality of the researcher was noted and revisited throughout various stages of the research process in order to capture initial reactions and emerging themes. The continuous revisiting of one's positionality (being both an insider and outsider to this community) maintains trustworthiness in the research outcome (themes) by grounding it within the subjective perspectives and viewpoints of the services providers while also avoiding investigative bias (Maher et al., 2018; Nowell et al., 2017). The three first authors analyzed the transcripts independently and conducted a process of peer debriefing to ensure consistency, and obtained consensus in the analysis and interpretation before sharing it with three senior supervisors for review and feedback. The findings were relayed back to several participants to ensure resonance. Member checking (Sandelowski, 1986) was also carried out by presenting findings of this study to both South Asian and other racialized audiences living across Toronto in multiple community forums. All youth and adults expressed that the findings resonated with their life experiences. Moreover, while some time has passed since this data was initially collected, the researchers have continued member checking with mental health service providers and South Asian youth in Peel Region since data collection, who assert that the issues raised in these key informant interviews still remain as pressing as ever. While the COVID-19 pandemic has placed a greater spotlight on the urgent need for accessible mental health care and many are concerned over the long-term consequences of online schooling and isolation felt by youth during the pandemic, the concerns raised by participants in this study still remain relevant and the gaps in South Asian youth mental health care still need to be addressed.

### Interviews and focus groups

A semi-structured in-depth interview / focus group guide was developed to investigate the study's research questions, using Harrell and Bradley's (2009) interview guide. The interview asked participants about the following topics: pathways to care, mental health and recovery, barriers to and facilitators of seeking mental health care, knowledge of the mental health system and services, stigma, mental health care experience, current practices, and recommendations. All participants in this leg of the study opted for interviews in English. Interviews lasted on average an hour and a half and were audio-recorded. The mp3 files were transcribed verbatim for analysis.

### Data analysis

Thematic analysis (Braun & Clarke, 2006, 2013) was used to identify, analyze, and report patterns within the interview transcripts guided by the research questions. Coding was based on the research questions of interest. A constant comparative technique and iterative refinement process were used throughout. Qualitative analysis was done manually, and codes were organized into subthemes and overarching themes. Themes or patterns related to the five research questions outlined above were searched for in the transcripts. A thematic map was finalized, and detailed analyses were written for each theme.

### Ethics

Research ethics were submitted and approved by the Centre for Addiction and Mental Health's Research Ethics Board. Written informed consent was obtained from all participants and verbal consent was additionally obtained prior to audio recording the interviews. To ensure confidentiality, participants were asked to only use their first names in the discussion and not to use any identifying information. After transcription, interview data was anonymized by removing all names from transcripts.

### Funding

This research was supported by a strategic training grant from the Canadian Institutes of Health Research – Strategic Training Initiative in Health Research (CIHR-STIHR) provided for the Social Aetiology of Mental Illness (SAMI) Training Program at the Centre for Addiction and Mental Health (CAMH) and the University of Toronto.

## Results

### Mental health service provider sample profile

A sample of 22 frontline mental health service providers participated in this study (Table 1). Data saturation was achieved with this sample of service providers. Nine mental health service providers participated in one-on-one interviews, representing the following professions: psychiatrist (n  =  1), mental health practitioner (n = 1), social workers / youth counselors (n  =  4), peer counselor (n  =  1), school guidance counselor (n = 1), and youth substance abuse counselor (n  =  1). In addition, two focus groups (n  =  13) were carried out with social workers working primarily with children (n  =  7) and social workers working with youth with severe mental illness (n  =  6). Over 70% of the participants were women (16 out of 22).

**Table 1.** Sample demographics of frontline service providers.

Sex	Occupation / job title	Company/organization	Years of experience working in Peel Region
*Individual interviews*			
Female	Social worker	Culturally specific mental health services	1.5
Female	Hospital youth trauma social worker	Large city hospital	10
Male	Psychiatrist	Private practice	6
Female	Researcher, mental health practitioner	Large mental health hospital, private practice	4
Female	Youth program coordinator	Regional youth mental health services	10
Female	Counselor	Place of worship	2
Female	Youth worker	Youth Substance Abuse Program	9
Female	Peer phoneline youth mental health support worker	Culturally specific youth mental health phoneline	1
Female	Social worker, guidance counselor	Culturally specific elementary school	4
Male	Motivational counselor	Place of worship	3
*Focus groups*			
3 females; 4 males	Social workers	Regional children’s social services	5–13
5 females; 1 male	Social workers working with youth with severe mental illness	Specialized youth mental health services	1–10

The same semi-structured interview protocol was used for both interview and focus group sessions. Thematic triangulation of focus group and interview data was pursued in this study to achieve analytical richness (Denzin, 1978). When transcripts were analyzed, the themes that emerged from the interview and focus groups converged. Therefore the results are discussed together.

This study is unique in that the majority of participants (over 75%) were from South Asian populations themselves and most worked with South Asian youth within community settings. All service providers practiced in Peel, and some had experience working in other parts of Toronto, such as Scarborough, Orangeville, and Etobicoke. Some service providers were practicing in multiple locations at the time of the interview. The participants ranged from having 1.5 to 30 years of experience working with South Asian youth (median 7.5 years; SD 7.1).

### Major themes

**Figure 1.**
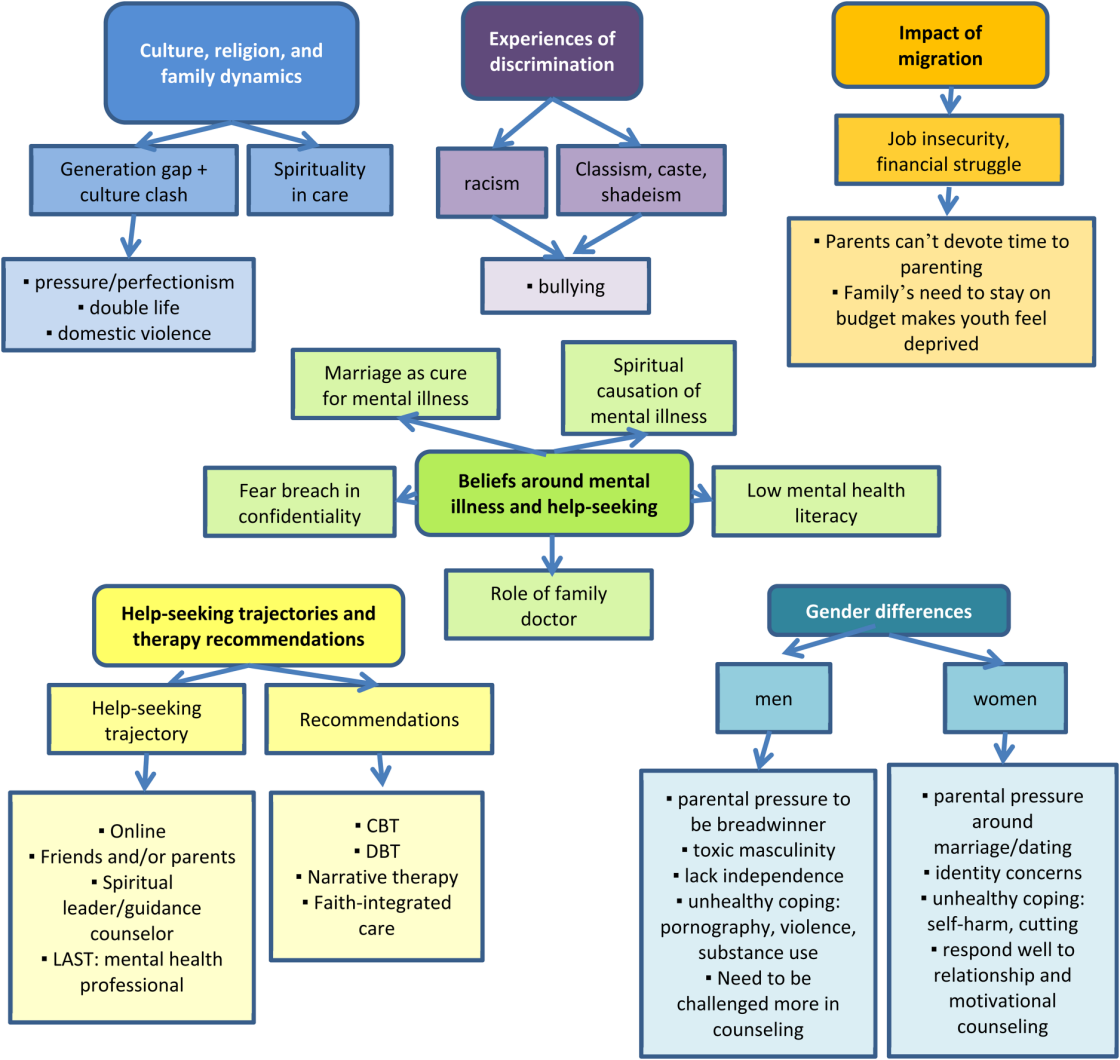
Thematic map of six major themes put forth by service providers as key areas of uniqueness to consider when working with South Asian youth mental health.

Participants outlined six areas of uniqueness that mental health professionals need to be aware of to better serve South Asian youth populations: A) South Asian culture, religion, and family dynamics, B) experiences of discrimination, C) impact of migration, D) beliefs around mental illness and help-seeking, E) help-seeking trajectories and therapy recommendations, and F) sex differences. The thematic map is summarized in Figure 1.

Mental health service providers pointed out barriers and had recommendations for all levels of society, covering systemic and structural issues, as well as family- and community-level barriers. Those who worked within a mainstream hospital or clinical setting often had more systemic-level barriers to share, while those who were community mental health workers had a rich understanding of family-level barriers. No level was more emphasized than others, and participants did not place blame upon youth. Rather, a systems approach that requires a holistic and multi-level effort to ensure the mental health of South Asian youth was discussed in all interviews.

#### Culture, religion, and family dynamics

According to service providers, South Asian youth growing up in Canada face not only a generation gap but also an added layer of cultural clash at home. As one mental health practitioner stated, “First generation parents … just want to keep their family together, like a fear of their children assimilating.”

South Asian parents may use parenting styles and discipline techniques, encouraging dependence and obedience. Service providers shared that the constant comparison, judgment, and perfectionism are sources of stress for youth.

… culturally, South Asian youth have a lot more pressure from their parents in terms of academics, just that perfectionism is kind of required. So yes, I get a lot of kids coming in with anxiety [and] some of them coming in with depression. They just don’t know how to organize their schedule and they feel overwhelmed. (School guidance counselor)

Service providers recounted clients who struggle with leading a “double life”—one life that is amenable to their parents and another to their peers. This split identity is the source of conflict and guilt for many youth. As one provider shared, “Parents want to impose their own values while youth here have to face a completely different culture/atmosphere in school … causing a clash between the youth and parents.”

[Youth] lose their autonomy, [they feel] they are losing their identity … and are struggling to recapture [their identity] and yet be respectful of their traditions and the parental role. But we also have [youth] who were angry with their parents, [who they feel are] very intrusive of their privacy, their autonomy. (Focus group, youth mental health social worker)

Several participants also noted that intimate partner violence (domestic violence) is prevalent in South Asian families and care needs to be taken to identify this stigmatized issue. One school guidance counselor recounted, “… a higher number of [my clients] were South Asians and there was a lot of domestic violence.”

It was interesting to note that regardless of participants’ own religious affiliations, the vast majority of mental health service providers emphasized the importance of faith-based coping and spiritualty for South Asian youth and the need to incorporate spiritual practices into therapy. The majority of participants also recommended that mainstream and community mental health organizations need to build bridges with faith leaders and places of worship in order to destigmatize mental health and bring services into South Asian communities: “Especially for South Asians who are very spiritual, why not get them to attend the spiritual center and help them there? If that is their comfort zone? That's huge. Our spiritual leaders and whatnot they don’t do that” (Social worker).

#### Discrimination

Service providers were mixed on the experiences of racism by South Asian youth in Peel. Several participants felt that because of the high percentage of South Asian populations in Peel, South Asians did not experience racism, but rather faced issues such as class and caste discrimination and shadeism within their own communities. Shadeism is discrimination based on skin color, usually within one's own race. This discrimination sets up a dichotomy of “darker” vs. “lighter/fairer” skin color, disadvantaging South Asians with darker skin color, and is rooted in a long history of anti-blackness, colonialism, and class discrimination. These issues can contribute to mental health issues, such as bullying, as recounted by one guidance counselor:

For example South Asian youth that are maybe from the northern areas like Afghanistan may not face the same issues as, say, a child who's from Bangladesh … They do often face prejudice based on the color of their skin … where they are from … the language that they speak or what they don’t speak. (School guidance counselor)

On the other hand, several service providers felt that in addition to this discrimination, South Asian youth in Peel also faced racism from the larger non-South Asian population. Participants recounted that many South Asian youth face discrimination and are bullied for being racialized as South Asian or a religious minority, particularly if they engage in visible religious practices (e.g., wearing a turban or hijab) and that this often manifests in the young person as anger or aggression.

Our South Asian population in Peel Region is large [in] number, but in our schools we still face kids wearing hijabs or turbans being bullied … Having the turban pulled off by other kids and the teacher not understanding why that is an issue … Kids bullying the girls to take off the hijab to the point where the kids feel like you know “it might not be safe for me to wear that” … Parents think “Why aren’t you wearing it?” and the girls would be changing in and out of [their hijab at] school. The line of communication, I find, is very severed sometimes. (Focus group, children's mental health social worker)

#### Migration

The interlocking structures of immigration status, race, class, and sex all intersect, compounding the oppression experienced by South Asian youth. Participants observed that the struggle to secure economic integration means that new parents lack support, often work multiple jobs, and are unable to devote time to raising their children: “I think that for immigrant parents they’re dealing with a lot … they only know what they know at that point, based on their own limitations … that impacts on their parenting, their decisions, and their availability” (Mental health practitioner, private practice).

Moreover, the struggle to secure economic integration often leads to feelings of powerlessness for parents, which manifests as impossibly high expectations. South Asian youth struggle to find their own identity amid the crushing weight of these expectations and their parents’ need to project a certain image of “success” to their families back home.

Family played a huge part in the stress that triggered some of the depression and anxiety, especially around high expectations. Parents were often first generation here in Canada so the young people … really felt that added pressure you know, “My parents sacrificed their life,” “They worked as a cab driver,” or “They gave up their dream so I have to fulfill that,” and that puts a lot of weight on an individual at that age when they are trying to figure out who they are just in terms of their developmental stages. They’re trying to figure out who they are in this world. They have this added layer of you know my parents want me to be this person. Another piece I found kind of interesting … particularly [for] males, the pressure of what people back home were also thinking. So extended family, whether it’s grandparents, uncles, aunts. They were the family that moved to the Western world and there's a particular image that's meant to be maintained around that. (Mental health practitioner, private practice)

Some participants mentioned that the immigrant outlook on saving money, even when economically secure, can be challenging for youth, who feel deprived when they compare themselves to their peers:

… some of the parents are sort of okay financially … But that back home mentality that you have to save every penny … we have to save, we don’t have enough! … It really, really affects [youth]. It's bad. They don’t go out anywhere. They don’t get to go on school trips or get anything. (Mental health counselor at place of worship)

#### Beliefs around mental illness and help-seeking and knowledge of mental health

Mental health service providers felt that certain beliefs regarding mental illness and help-seeking may make accessing care challenging for South Asian families. The belief that marriage was a “cure” for mental illness, especially for young women, was one concern that participants raised:

[The community] will preach marriage as the ideal and the solution to everything … I feel that healing does not exist as a term sometimes … there are just too many quick fixes and not trying to understand what is actually going on here. (Peer counselor, cultural-specific mental health helpline)

Parents are hesitant to seek help from mainstream services, fearing breach of confidentiality. They reported that families are often concerned about projecting the image of perfection and reluctant to seek care because a diagnosis may sabotage their child's chances for “success.” This was also gendered, with parents fearing their daughters would not marry and their sons would not find employment. One mental health counselor at a place of worship described how some parents, after hearing their children are seeking counseling, feel the need “to start hiding”: “[They fear their children] will change … [that the counselor will] turn [their children] against [them]—those are the fears. And a fear of … telling personal stuff. They don’t perhaps understand confidentiality” (Mental health counselor, place of worship).

Some youth and parents may also be distrustful of Western psychiatry and the use of psychotropic medication. South Asian families may hold beliefs of spiritual causation of mental illness and attempt traditional healing or seek treatment out of the country. One provider recounted a client who told him that “psychiatric medications are a tool of the *jinn* [spirits in Islamic spirituality].”

Service providers noted that if the youth is able to access care, they will often encounter further barriers, such as parents’ dismissal of the diagnosis. The family may be unable to recognize the mental health issue as a chronic condition that needs ongoing management and see it as a result of common stressors that are part of adolescence (e.g., academic pressure) instead.

… parents don’t agree with counseling; they ask, “What's all this talk of feelings and mental health?” [Youth] would love to come [for counseling] but they would have to hide from their families and lie to them so it is difficult. (Mental health counselor, place of worship)

If the family accepts the diagnosis, they may catastrophize by focusing on the illness and be unable to envision recovery. For example, one hospital youth trauma social worker said she was once asked by a mother, “Does it mean my child is crazy?” The parents may insist that mental health professionals “fix” their child and not take part in a collaborative recovery process. Service providers reported that the underdeveloped mental health system and lack of mental health awareness in the family's country of origin also present problems, as the family is unaware of family history of mental illness or reluctant to disclose family issues. Community stigma and silence over domestic violence and the overlay of toxic masculinity with alcoholism in South Asian families also presents challenges.

Service providers suggested that one area of opportunity for mental health intervention is the deference South Asian parents have for doctors. Psychoeducation and mental health resource linking provided by the family doctor can act as a potential area of intervention. Another key area of uniqueness for South Asian youth is their connection with their family and spirituality. Service providers recommend that youth be reconnected with their family and faith as a part of the recovery process. Participants felt that liaisons with the South Asian community and mental health organizations could help to break down mental health stigma. Mainstream services can foster respect for South Asian understandings and co-create mental health resources. Community and faith spaces are often on the frontlines of mental health work, and in the absence of culturally safe models of care from mainstream organizations, community spaces have taken the brunt of serving the community's needs without the funding and support mainstream organizations receive. Rather than working in silos, mainstream mental health organizations can build trust by building bridges into areas where the community congregates.

So we have to think of where do families go for help before they come here [to mainstream services]? Before they go to a doctor, where do they go? Coz if [mainstream services] are going to be respectful of [South Asian] religious leaders and community leaders, then you’ve got to start thinking of going into the community and bridge-building somehow … offer to go in, start exploring what they are telling families … what they are offering. (Focus group, youth mental health social worker)

Lastly, service providers identified the lack of mental health literacy and knowledge of mental health resources available in Peel as a significant barrier. Service providers in the individual and focus group interview rated the average level of mental health resource knowledge of South Asian youth on a scale of 1 to 5 (1 being no knowledge at all and 5 being they are experts), and rated youth at a poor level of knowledge with an average rating of 1.73 (SD 0.95).

#### Help-seeking trajectories and therapy recommendations

Service providers lent their insights on the varying trajectories that South Asian youth take in seeking mental health care. All service providers agreed that most youth first seek mental health information online (e.g., Google, blogs / video blogs, YouTube, social media, etc.) and then go to their peers for advice.

Although all agreed that youth used online information sources, younger service providers were more likely than older respondents to recommend building on this tendency by developing social media and online mental health tools to reach youth.

[To find mental health information, youth go] Online. They try to self-medicate. They try to manage their depression through Googling and online because they don’t want to talk to other people about it. They want control over it. I find. So making sure that we have good information online [is important]. (Focus group, children's mental health social worker)

After searching online, youth vary in their trajectories. Some may confide in their friends or parents, others may seek counsel from a spiritual leader, while others go to the school counselor. Much later, youth may arrive at the doors of a community or mainstream mental health organization or professional's office. Generally, service providers felt that South Asian youth took anywhere from six months to a year to finally seek professional mental health care.

[Youth] usually don’t go to anyone and suffer on their own, alone … The next step, if they have the confidence to approach their peer group … Then they may approach a family friend or a parent … Some may go to the imam [Muslim spiritual leader] at the masjid [mosque] … They don’t usually find that as useful because they already heard the same thing from their parents. That is what my clients say. (*laughs*) And then … someone will tell them, there is counseling being offered at [the mosque], then they will come to me … But I am usually the last person to know, the last person they get to. (Mental health counselor at place of worship)

Mental health service providers also mentioned additional trajectories. Sometimes a precipitating event served as a wakeup call for parents, such as their child being involved in a crime, or failing or dropping out of school. The parents sometimes opted to first seek advice from the family doctor rather than go to specialized mental health services. Other times, the mental health issue led to a point of crisis (e.g., suicide attempt or psychosis) which then finally provided the impetus for the family to take their child to the emergency department to seek mental health care. Service providers lamented the poor prognosis associated with emergency department visits, which can often be traumatic and impede continuity of care following hospital discharge.

Service providers had varied recommendations for therapy modalities when working with South Asian youth. Some participants felt that Cognitive Behavioral Therapy (CBT) was useful in helping youth brainstorm and problem solve. Others felt that CBT and Dialectical Behavioral Therapy (DBT) may work for South Asian youth but family therapy work also needs to be included: “I think systemic family therapy really works; I find that working with South Asian families, youth, CBT, DBT are really useful to reduce mental health symptoms and harmful behaviors but you also have to do some family work” (Youth program coordinator).

The inclusion of religiously integrated care was also recommended, as faith-based coping is important to South Asian youth. Lastly, several participants felt that CBT was not helpful for South Asian youth as it places too much emphasis on the individual, invalidates emotions/experiences, and silences personal stories. Therapeutic approaches such as narrative therapy and mindfulness were emphasized instead, for their ability to empower youth. As one hospital social worker stated, “Narrative takes a long time but it provides context and background which is crucial.”

#### Sex differences

From their experience, service providers identified key differences in the mental health concerns that young South Asian men and women face. While 72% of the sample were women, both men and women spoke about the importance of considering South Asian constructions of gender roles and the different stressors they place on young South Asian men and women.

I think there is a bit of difference in terms of challenges faced by men and women. I feel like most of the females are associated with a relationship issue and how to navigate those … [for example] arranged marriage or love marriage. For boys, it is substance-related [issues] that … [impact their] mental health and wellbeing [and may be used as a] coping strategy. (Focus group, children's mental health social worker)

According to service providers, young South Asian women were more likely to be concerned about their identity, faced culture clash and family conflict over gender roles and expectations for young women, and faced extreme pressure and parental regulation in regards to marriage. However, the extent to which young women faced these pressures varied widely as a result of other social identities, reflecting the importance of maintaining an intersectional lens. As one social worker in the focus group stated, “Some enter into a marriage, [e]specially … young South Asian females. There is a lot more pressure on them, depending on culture and religiously.” As many South Asian populations come from cultures and faith backgrounds that emphasize abstinence before marriage, there is a strong push to get married young.

In contrast, the major source of parental clash for young South Asian men usually occurred over academic and career success, facing extreme pressure to be a “success” and to take on the “breadwinning role and support the family” and live up to “that stereotype of what masculinity is,” as one social worker who works with children stated during a focus group. At the same time, service providers felt that young South Asian men were coddled at home, particularly by their mothers, and conditioned to be overly dependent. One mental health practitioner described how young South Asian men struggle with a sense of “entitlement” where they have grown up “being allowed to kind of do what they’re doing, no limitations no sanctions, no repercussions, no punishment.” One participant during the focus group with social workers working with youth living with severe mental illness expressed how some young men “struggle with expressing their independence … because as a boy [they] never had to do … laundry or cook for [them]selves.”

A few service providers emphasized the need to start early intervention, helping parents to nurture the attachment and mental health of infants and children to mitigate youth mental health issues down the road. Several participants also stressed the need to address the poor communication between parents and children within South Asian families. Communication breakdown often occurred around gender roles and parental control, where young women clashed with parents over dating/marriage and restrictions on their social life, while young men were fed messages of toxic masculinity which socialized them to believe that it is not masculine to communicate emotions or feelings. In homes struggling with alcoholism and binge drinking, young South Asian men dealt with toxic masculinity and negative role modeling from men in the family. Often, young South Asian men turned to unhealthy coping mechanisms (e.g., substance use, violence, or pornography) to self-medicate and numb the pain of their struggles. Young South Asian men were less likely to confide in someone about their mental health issues.

Youth often [talk to] their friends. Boys, however, are more closed. Oftentimes they turn to alcohol and drugs to numb the issue. This is generational and systemic. Males in the family often engage in drinking and binge drinking or drugs. This is common for South Asian males. They’ll say, “Well, my dad and uncle drink. Why can’t I?” There is a real need for education here. (Hospital youth trauma social worker)

In contrast, young South Asian women were more likely to engage in self-harm (e.g., cutting) to deal with their pain. In regards to therapeutic practice, service providers felt that relationship and motivational counseling work well for young South Asian women, while young South Asian men need to be pushed more and challenged outside their comfort zones during counseling.

## Discussion

This study presents mental health service providers’ perspectives on the barriers South Asian youth face in trying to access mental health care in Peel Region, Toronto, Canada. In particular, the study findings reflect unique insights from service providers of South Asian background, many of whom are trying to bridge siloes between mainstream mental health services and the culturally specific mental health needs of diverse South Asian communities. According to mental health service providers, South Asian youth navigate a number of unique stressors related to the domains of culture, religion, and family dynamics, experiences of discrimination, the impact of migration, beliefs around mental illness and help-seeking, help-seeking trajectories and therapy recommendations, and lastly, sex differences. The steps needed to effectively address the unique mental health challenges, best practice guidelines, and recommendations for working with South Asian youth, families, and communities were outlined to provide a practical, nuanced, and a frontline-based overview on how mental health care can effectively meet the needs of South Asian youth populations.

Service providers identified an array of mental health service access barriers experienced by South Asian youth at the levels of family, community, and the mental health system. These echoed the views of the South Asian youth interviewed in another branch of this project (Islam et al., 2017). Youth reported systemic barriers such as long wait times, prohibitive fees, and the lack of culturally safe mental health care provision, in addition to the family-level barriers of mental health stigma and lack of mental health literacy. In line with the findings of this study, a study among South Asian youth living in the US also found that mental health stigma and fears around breach of confidentiality were major barriers (Rastogi et al., 2014). South Asian American youth also expressed that “helicopter parenting,” where parents micromanage their children's lives, prevented young people from seeking help as they feared their parents would take control of their therapy (Rastogi et al., 2014). A review of access barriers experienced by South Asian families and youth in the US found similar barriers to those uncovered in this study. Sharma et al. (2020) reported that the mental health stressors associated with acculturation and the pressure of being a part of a “model minority,” mental health stigma, and the need to sensitively navigate the parental and youth generations in South Asian families were important to consider when working with South Asian populations. Culturally adapted family therapy and community-based approaches were recommended to best engage South Asian youth and their families in mental health care, which echoed many of the recommendations put forth by the key informants in this study. The multiple interlocking structures of discrimination and stressors of migration, mental health, and South Asian identity were a key factor under investigation in this study. South Asian youth living in South Asia were bullied most often by peers of their own cultural group for differences in caste and class (Craig et al., 2009; Rahman et al., 2020). South Asian youth in Peel faced this type of bullying in addition to being bullied by peers outside of their cultural group, for example for wearing a turban or hijab. South Asian populations in South Asia faced many of the same barriers to mental health service access as South Asian youth in Peel Region, such as mental health stigma, poor interpersonal support, and difficulties in accessing mental health services (e.g., unable to commute for therapy) (Aggarwal, 2012). The preference to seek alternatives to medical/psychiatric care was also observed in South Asia, with people opting for community and/or spiritual therapy (Rahman, 2007; Wasan et al., 2009). In this study, most South Asian youth also went through several care options (e.g., online, peers, trusted adult, school counselor, spiritual leader) before seeking specialized mental health care. Programs to decrease mental health stigma, increase mental health service access (for example through the use of mobile technology), and increase mental health literacy (Aggarwal, 2012) could all benefit South Asian populations living in South Asia as well as in the diaspora.

In addition to the mental health challenges that all youth face, service providers identified key areas of uniqueness for South Asian youth in the domains of culture (e.g., arranged marriage), religion, and experiences of discrimination (e.g., caste-based discrimination and shadeism specific to the South Asian context), family dynamics, the impact of migration, and beliefs around mental illness and help-seeking. South Asian youth interviewed in an earlier leg of this study also identified acculturative stress and intergenerational conflict as major sources of stress in their lives (Islam et al., 2017). South Asian youth living in the U.S. described cultural clash and intergenerational conflict as major mental health stressors, especially in the domains of dating and academic success (Rastogi et al., 2014). In Asian families, family duty and pressure are often emphasized over autonomy so that the family can function as a unit, and this can lead to conflict between parents and youth (Ekanayake et al., 2012; Tran et al., 2005). Service providers in this study went into great detail about the sex differences in mental health concerns experienced by South Asian youth, which were often connected to differing gender roles and expectations. The use of tools such as the Cultural Values Conflict Scale (Inman et al., 2001) may help clinicians and service providers better assess the stress that South Asian youth undergo as they try to negotiate gender role expectations.

South Asian immigrants struggling to achieve economic integration in Canada face increased intergenerational tension and isolation when their children begin to adapt to the new culture and become strangers to them (Islam et al., 2020, 2021; Shariff, 2009). There is strong evidence that compounding layers of structural barriers and discriminations (racism in hiring processes, non-recognition of international credentials, language barriers, information barriers, limited social connection and social support system) prevent racialized and immigrant communities in Canada from obtaining well-paid, meaningful employment in their field. As documented in our study, the powerlessness of under/unemployment often leads parents from immigrant and racialized backgrounds to try to control their children to hold onto what bit of power they have left. Parents living out their unrealized dreams through their children place extreme pressure on children to succeed according to parental expectations. In addition, working multiple jobs to make ends meet does not allow for optimal parenting, which can compound and contribute to youth mental health issues. This cultural clash and intergenerational conflict, which is the major stressor for South Asian youth (Islam et al., 2017), could be mitigated if the barriers to economic integration were reduced for migrant communities, for example through government programs like job-matching, skills-bridging, and affordable childcare (Access Alliance Multicultural Health and Community Services, 2012; Premji & Shakya, 2017; Wilson, 2011) and by offering culturally safe early childhood education and parenting support programs (Karoly, 2010; Mental Health Commission of Canada, 2013).

Results from this study show that service providers from South Asian backgrounds, particularly those based in community settings, appear to have in-depth knowledge about mental health stressors, needs, and service barriers facing South Asian youth, including insights about how to make mental health services more culturally safe. Policy/decision makers within the mental health sector, including those responsible for developing new interventions, need to consult with service providers who work closely with vulnerable communities and/or are from within vulnerable communities themselves. Many of the participants were providers in community spiritual centers or services that integrated mental health and spiritual care together. Organizations like these can act as important bridging sites. Working synergistically, mainstream mental health organizations with their funding and resources can partner with community and spiritual organizations, which are providing frontline mental health care and have built trust within South Asian communities. They can work together to provide care for South Asian youth populations. Service providers emphasized the need to develop culturally safe models of mental health care and, regardless of their own personal religious beliefs, recommended that spiritually integrated, holistic mental health care may be the most effective for South Asian youth. The development of culturally safe models of mental health care is also recommended in Canada's Mental Health Strategy (Mental Health Commission of Canada, 2015). Rathod et al. (2010) found that CBT can be impactful for racialized communities when adapted to be culturally appropriate and specific to patient needs. Religious adaptations of CBT have been developed (Haque et al., 2016; Pargament et al., 2000; Pearce, 2015) and, more specifically, CBT has been adapted to meet the needs of South Asian Muslim populations (Naeem et al., 2015). In the present study, one service provider noted that South Asian youth often felt that their emotions/experiences were invalidated by CBT. Recommendations were given for greater space to share personal narratives within CBT and for consideration of the interdependent nature of family life especially for South Asian youth, rather than focusing solely on individualistic behavioral change and goal-setting. Cultural adaptation of CBT that incorporates elements of empowerment, narrative therapy, and mindfulness may be beneficial in working with South Asian youth.

Considering the common conflicts that South Asian families experience at home, counseling techniques aimed at increasing communication and empathy between parents and youth may be helpful. Furthermore, to provide culturally safe care suited to South Asian youth populations, sensitizing therapy to conflicts experienced through the acculturation process, adolescent ethnic identification, parenting stress, and the roles and expectations placed upon different family members needs to be considered (Shariff, 2009).

The impact of migration on youth mental health as a result of acculturation and the stressors of the migration and resettlement process begins long before adolescence. Service providers recommended that an early childhood mental health promotion approach be taken. In order to effectively prevent youth mental health issues, the work needs to start from birth. Parents need to be offered mental health education at the hospital post-birth to foster infant mental health care and positive parenting. The Triple P – Positive Parenting Program® has been recommended by the Public Health Agency of Canada as an evidence-based best practice intervention for parents of young children to adolescents (Public Health Agency of Canada, 2013), and has been adapted for racialized and low-income settings (Houlding et al., 2012; Public Health Agency of Canada, 2013; Sanders et al., 2014; Triple P Canada, 2021). Based on the recommendations from this study, developing a culturally safe adaptation of the Triple P – Positive Parenting Program® tailored towards South Asian families in Canada, which incorporates modules on intergenerational conflict, cultural clash, faith-based coping, and other unique factors, could be a powerful mental health intervention for South Asian families.

Future research can carry out program evaluations of mental health services that tailor their programming for South Asian youth in Peel Region using the best practices and recommendations outlined in this study. It would be important to understand if such tailored programming is more effective, has lower dropout rates, and higher rates of recovery. Studies on the best methods for bridging between mainstream mental health services and culturally specific or faith-based organizations doing frontline mental health work would help to advance the field. Future research with South Asian families of youth in mental health services to understand the barriers they faced and the recommendations they have for improvement would also be helpful.

This study has a number of limitations. This was a qualitative study of service provider perceptions, which may be susceptible to recall bias. However, triangulation of information was carried out with findings from the research project's previous phase with South Asian youth (Islam et al., 2017), as well as through using both individual interviews and focus groups to gather data. The majority of the service providers identified as South Asian themselves and worked within community settings, and as a result, over-identification and personal interpretation of their clients’ experiences may have taken place. While cultural/linguistic/religious matching can be a facilitator of mental health care (Minnis et al., 2003; Rehman, 2007), it can also serve as a barrier when the client worries that there will be a breach of confidentiality that can lead to gossip and judgment from the community (Gulliver et al., 2010; Islam et al., 2020). In such cases, optimal therapeutic rapport and trust may not be achieved and the client may feel inhibited from openly sharing their experiences. However, the South Asian providers’ insider/outsider dual positionality allowed them a more nuanced framing to their insights. This insider/outsider aspect of the study is also a unique strength in that most of the service provider participants were from the communities that their clients were from (in terms of region of origin, culture, ethnicity, religion etc.) and working within community settings.

## Conclusion

Most studies focus on mainstream providers/settings and thus their key recommendations are limited to saying that services need to be made more culturally sensitive and highlighting the need for service providers from diverse backgrounds. However, this study goes beyond cultural appropriateness and covers mental health barriers that persist even when South Asian mental health professionals are providing care to South Asian youth within community settings (e.g., mosques, culturally/religiously specific schools, etc.). Studies like this lay the groundwork to uncover the deeper structural determinants and the complex intersectional relationship between these determinants that affect mental health and mental health care access. This article provides service provider insights on the need for a multi-level and culturally safe approach to counseling and mental health intervention that considers the unique stressors experienced by South Asian diaspora families living in cosmopolitan cities like the Peel Region of Canada.
